# Supplementary material for: Prediction of minimal hepatic encephalopathy by using an radiomics nomogram in chronic hepatic schistosomiasis patients
Source: PLoS Negl Trop Dis. 2021 Oct 15;15(10):e0009834. doi: 10.1371/journal.pntd.0009834 (PMC8550421; doi:10.1371/journal.pntd.0009834)
Supplement: S1 Table — (DOCX) [file pntd.0009834.s001.docx]

S1 Table. MRI examination's parameters.

| Sequences | Parameters |
| --- | --- |
| T1WI | VIBE,TR/TE=3.4/1.3, Matrix=320×320, thickness=3mm, FOV=260×260mm |
| T2WI | TSE,TR/TE=2770/64, Matrix=320×320, thickness=4mm, FOV=260×260mm |
| DWI | EPI,TR/TE=7100/79, b=800, Matrix=256×256, thickness=4mm, FOV=260×260mm |

DWI: diffusion weighted imaging; EPI: echo planar imaging; FOV: field of view; T1WI: T1-weighted imaging; T2WI: T2-weighted imaging; TE: time of echo; TR: time of repetition; TSE: turbo spin echo; VIBE: volumetric interpolated breath-hold examination
