# Supplementary material for: Prediction of minimal hepatic encephalopathy by using an radiomics nomogram in chronic hepatic schistosomiasis patients
Source: PLoS Negl Trop Dis. 2021 Oct 15;15(10):e0009834. doi: 10.1371/journal.pntd.0009834 (PMC8550421; doi:10.1371/journal.pntd.0009834)
Supplement: S2 Table — (DOCX) [file pntd.0009834.s002.docx]

S2 Table. Logistic regression analyses results of clinical predictors

| Features | Estimate | Std. error | t | P |
| --- | --- | --- | --- | --- |
| Intercept | 3.56 | 0.59 | 6.01 | < 0.001 |
| Age | -0.01 | 0.00 | -1.43 | 0.156 |
| ALT | 0.00 | 0.00 | 0.73 | 0.465 |
| AST | 0.00 | 0.00 | 1.55 | 0.123 |
| TB | 0.00 | 0.00 | -0.16 | 0.874 |
| PT | -0.01 | 0.03 | -0.48 | 0.631 |
| UB | 0.00 | 0.01 | -0.34 | 0.736 |
| Albumin | -0.01 | 0.00 | -3.48 | 0.001 |
| Plasma ammonia | 0.01 | 0.00 | 3.56 | 0.001 |
| Platelet count | -0.01 | 0.00 | -6.48 | <0.001 |

Alanine aminotransferase (ALT), aspartate aminotransferase (AST), total bilirubin (TB) prothrombin time (PT), unconjugated bilirubin (UB)
