# Supplementary figures and images for: Prediction of minimal hepatic encephalopathy by using an radiomics nomogram in chronic hepatic schistosomiasis patients
Source: PLoS Negl Trop Dis. 2021 Oct 15;15(10):e0009834. doi: 10.1371/journal.pntd.0009834 (PMC8550421; doi:10.1371/journal.pntd.0009834)

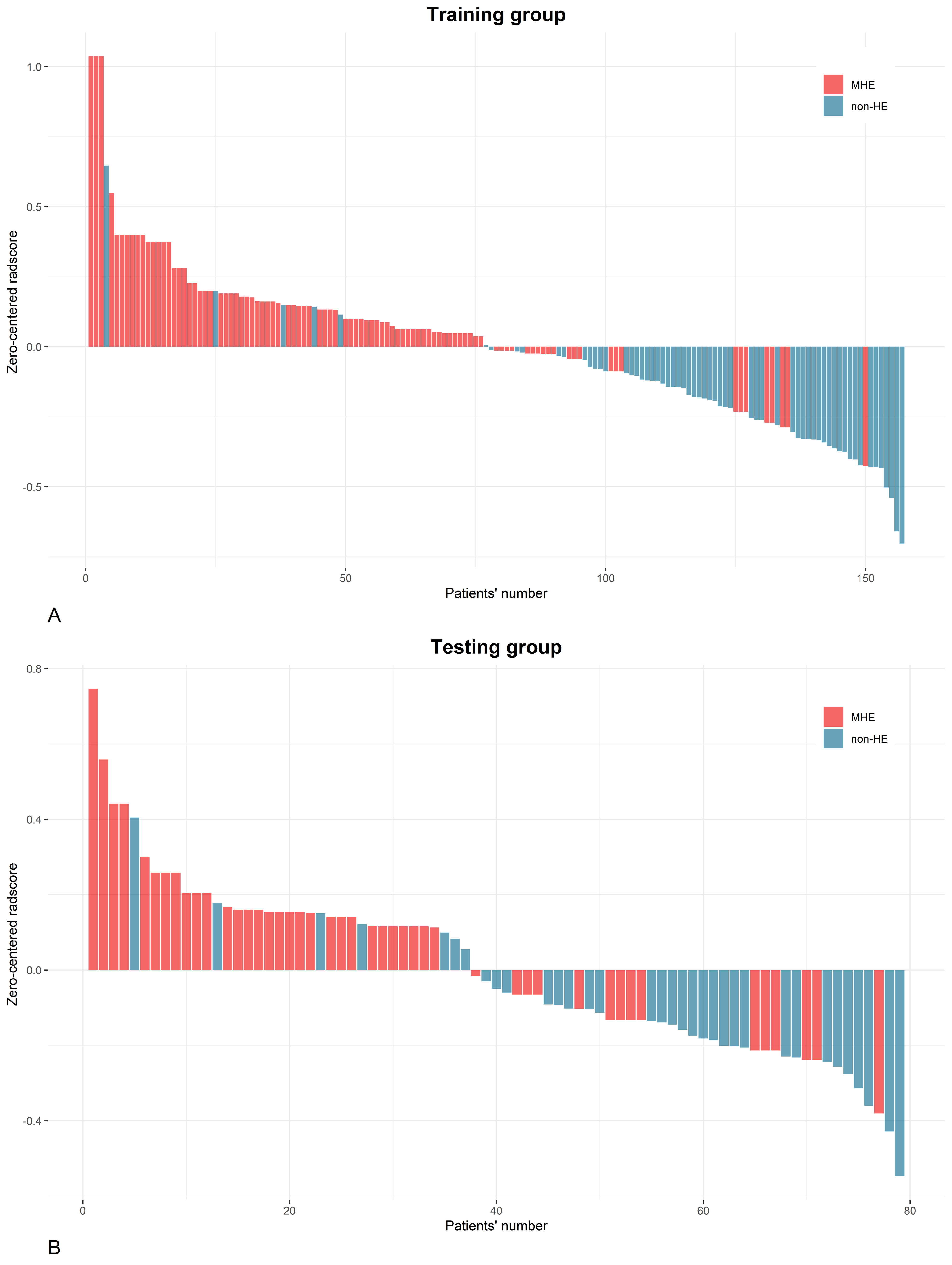

Supplement: S1 Fig — The radscore of each CHS patients with MHE (rad bar) in the training group (A) and testing group (B). CHS, chronic hepatic schistosomiasis; MHE, minimal hepatic encephalopathy. (TIF) [file pntd.0009834.s003.tif]
