# Supplementary material for: Prediction of minimal hepatic encephalopathy by using an radiomics nomogram in chronic hepatic schistosomiasis patients
Source: PLoS Negl Trop Dis. 2021 Oct 15;15(10):e0009834. doi: 10.1371/journal.pntd.0009834 (PMC8550421; doi:10.1371/journal.pntd.0009834)
Supplement: Attachment — Submitted filename: Response.docx [file pntd.0009834.s004.docx]

Dear Editor Matty Knight and Michael Hsieh,

Thank you very much for giving us an opportunity to revise our manuscript. Your comments and meaningful suggestions are all valuable and helpful for improving our paper to be suitable for publication. We have considered carefully these comments and made relevant corrections point-by-point.

Reviewer #1:

Methods

#1 The authors appeared to perform their analysis carefully. However, the evaluation does not appear to be thorough. The current work evaluated only the predicting performance of the combined features. It is difficult to state that integrating two feature sets is useful without proper comparison or analysis. The authors should have evaluated two feature sets (radiomic features and clinical risk factors) separately first. Then, the combined radiomic and clinical features could be compared to see any improvement in predicting performance. Ideally, clinical features vs. radiomic features vs. combined features vs. combined features with selection (reduced combined features that are reported in this manuscript) should have been analyzed to draw the authors' conclusion.

Response: Thanks for the suggestion. We have added the results of both clinical features and radiomics signature, and compared the predicting performance between these models. Results had been shown in Table 2.

Table 2 The diagnostic performance of the clinical features, radiomics signature and nomogram in the training and testing groups

| Group | Model | SPE | SEN | NPV | PPV | AUC (95% CI) | P* | P# |
| --- | --- | --- | --- | --- | --- | --- | --- | --- |
|  | Clinical features | 75.8% | 83.1% | 74.6% | 84.0% | 0.81 (0.75-0.88) | 0.138 | - |
| Training | Radiomics signature | 88.7% | 82.1% | 76.4% | 91.8% | 0.88 (0.82-0.94) | - | 0.138 |
|  | Radiomics nomogram | 90.3% | 86.3% | 81.2% | 93.2% | 0.91 (0.86-0.96) | 0.146 | 0.003 |
|  | Clinical features | 76.5% | 66.7% | 63.4% | 78.9% | 0.75 (0.65-0.86) | 0.780 | - |
| Testing | Radiomics signature | 88.2% | 66.7% | 66.7% | 88.2% | 0.77 (0.67-0.88) | - | 0.780 |
|  | Radiomics nomogram | 85.3% | 75.6% | 72.5% | 87.2% | 0.83 (0.74-0.92) | 0.084 | 0.071 |

AUC, area under the curve; CI, confidence interval; SEN, sensitivity; SPE, specificity; NPV, negative predictive value; PPV, positive predictive value.

* compared with radiomics signature; # compared with clinical features.

#2 The analysis needs to include more performance metrics, even if they may appear to be redundant. Machine-learning studies provide multiple performance metrics in general. In addition, conducting cross-validation would support the authors' methodology.

Response: Thanks for the question. We used binary least absolute shrinkage and selection operator (LASSO) logistic regression analysis to select the radiomics features. The radiomics features were calculated by LASSO with 10-fold cross-validation, and validated using testing data. We have explicated this in the text.

#3 Lines 108-110: The volume of interest is enough (or 3-D region of interest). This reviewer noticed a bit confusing use of the acronym of ICC. ICC is used to abbreviate the intraclass correlation coefficient. Interclass correlation coefficients are usually represented by their common names, e.g., Pearson's correlation coefficient. This reviewer recommends use ICC only for intraclass correlation coefficients. The current statement here appears to be ICC used for both intraclass and interclass correlation coefficients.

Response: Thanks for the suggestion. Both interclass correlation coefficients and interclass correlation coefficients were used to identify stable features. We do agree the abbreviation of ICC could bring confusion for the readers, and we have revised it in the text for more clear expression as suggestion.

#4 Line 116: Please, clarify 'unstable' features.

Response: Thanks for the question. Radiomics features with either intraclass correlation coefficients or interclass correlation coefficients < 0.75 were identified as unstable features. We have revised it in the text.

#5 Line 118: Please, clarify the term 'redundant'. Does this mean that a redundant feature strongly correlates to many other features simultaneously (for example., collinearity leading to variance inflation due to correlated independent predictors in a multiple regression model)?

Response: Thanks for the question. Redundant features mean that features with strongly correlates simultaneously. If features had a Pearson correlation coefficient > 0.9, the feature with the least mean absolute coefficient was retained. We have explicated it in the text.

#6 Lines 121-123: This is a potentially very important approach unless it has been published somewhere else. Its description appears to be similar to the concept of the latent variable from PCA (principal component analysis). The authors must spell out the "linear combination" approach used to created radscore. It is hidden in the current manuscript. If it has been published somewhere else, please provide its reference.

Response: Thanks for the suggestion. Radiomics score (radscore) for each patient was calculated using a linear combination of radiomics signature that were weighted by their respective coefficients. We explicated it in the text, and have added the reference[28].

Results

#7 Results section needs major revision.

The current result section is overly brief and needs more elaboration of tables/figures with quantitative information. It should not include descriptions appropriate for the methods section.

Response: Thanks for the suggestion. We have added the results of both radiomics and clinical features, and compared predicting performance between radiomics, clinical features and radiomics nomogram in the Result section.

Conclusions

#8 Current conclusion based on the presented methods and results did not convince this reviewer.

After having compared the two feature sets separately and combined, a more detailed discussion can be elaborated on the value and implications of integrating radiomics and clinical risk factors.

Response: Thanks for the suggestion. We have compared predicting performance between radiomics, clinical features and radiomics nomogram, and revised the Discussion section.

Summary and General Comments

#10 This manuscript is well organized and delivers an interesting application of a machine-learning methodology in a clinical study setting. The primary objective of this study is to evaluate a data-driven method developed to predict the minimal hepatic encephalopathy (MHE) in chronic hepatic schistosomiasis (CHS) patients.

Although the topic of the study will benefit clinicians and researchers in this field, the current form of the manuscript raised a few concerns. As such, this reviewer would like to encourage the authors to reflect comments and suggestions provided by this reviewer on the next iteration of the manuscript.

Response: Thanks for the suggestion. We have responded the reviewer's comments point-by-point .

Reviewer #2:

Methods

#1 Instead of removing the "redundant features" a dimension reduction algorithm could be an option.

Response: Thanks for the suggestion. Redundant features were considered as the features with a strong correlation with each other, which might lead to over-fitting in the radscore. If features had a Pearson correlation coefficient > 0.9, the feature with the least mean absolute coefficient was retained in this study. Thus, we did not use other dimension reduction algorithm to remove the redundant features. We have also explicated the method in the text.

#2 Why clinical features are handled by a separate model?

Response: Thanks for the question. The clinical features have be used to evaluate minimal hepatic encephalopathy in routine clinical practice. So the clinical features was handled by a separate model. And the adding value of radiomics to clinical features could be assessed. We have added the results of both radiomics and clinical features in Table 2.

#3 What is the contribution of the radiomics features to the model?

Response: Thanks for the question. We have developed the clinical features model and radiomics signature model according to your suggestion, and we performed the comparisons of predicting performance between clinical features, radiomics signature and radiomics nomogram to evaluate the contribution of the radiomics features. We have added the results in the Result section and made the relative discussion in the Discussion section.

#4 Which software has been used for the statistical analysis?

Response: Thanks for the question. All statistical analyses were performed in R (Version 4.0.2; <http://www.r-project.org/).> And all the packages listed in statistical analysis section were R packages.

#5 Are the authors applying the model developed in the training set to the ? From the results section it appears that the same features, not the same parameters have been applied.

Response: Thanks for the question. The same features developed in the training set were used in the testing set. And we tried to select features with a good robustness and reproducibility to acquire a stable diagnostic performance. We have explicated it in the text. Considering that different MR scanners and scanning parameters may be used, we think that the good robust and reproducible features would be better for generalization of the radiomics nomogram.

#6 The authors should predict what happen in the testing set using the model parameters from the training set.

Response: We do validated the model used the same features, not the same parameters. Because on considering the generalization of this study, we think it is appropriate to use the same features to validated the model. As we mentioned above that other studies may use different MR scanners, which would be unappropriated to use the same parameters, but they can use the same features derived from the radiomics. However, we also calculated the results of the testing set using the model parameters from the training set. A slight decrease was seen (model AUC by using the same features: 83.3% [95% CI: 0.74-0.92]; model AUC by using the same parameters: 82.0% [95% CI: 0.72-0.91]), but no significant difference was shown (by DeLong's test: D = 1.6021, df = 122.78, p-value = 0.1117).

Results

#7 The results need to be revised according to the new methods

Response: Thanks for the question. The results have been revised in the Result section.

Conclusions

#8 Some concerns on diagnostic nomograms developed with small sample size.

Response: Thanks for the question. We have added it in the Limitation.

We tried our best to improve the manuscript and made some changes which marked in red in revised paper. We thank Editors and Reviewers' excellent works and hope the modifications above meet your requests, and we trouble you to revise any errors in this paper. If you have any queries about this modified paper, please email to me (dr.jinweiqiang@163.com). Once again, thank you very much for your comments, suggestions and considering the publication of our paper.

Your sincerely

Jin Wei Qiang，MD.,PhD.

Professor and Chairman, Department of Radiology, Jinshan Hospital,

Fudan University, Shanghai, China
